# The Effects of Meditation on Perceived Stress and Related Indices of Psychological Status and Sympathetic Activation in Persons with Alzheimer's Disease and Their Caregivers: A Pilot Study

**DOI:** 10.1155/2012/927509

**Published:** 2012-02-21

**Authors:** K. E. Innes, T. K. Selfe, C. J. Brown, K. M. Rose, A. Thompson-Heisterman

**Affiliations:** ^1^Department of Community Medicine, West Virginia University School of Medicine, P.O. Box 9190, Morgantown, WV 26506-9190, USA; ^2^Center for the Study of Complementary and Alternative Therapies, University of Virginia Health System, P.O. Box 800782, McLeod Hall, Charlottesville, VA 22908-0782, USA; ^3^Department: Family, Community & Mental Health Systems, School of Nursing, University of Virginia Health System, Charlottesville, VA 22908-0782, USA

## Abstract

*Objective*. To investigate the effects of an 8-week meditation program on perceived stress, sleep, mood, and related outcomes in adults with cognitive impairment and their caregivers. *Methods*. Community-dwelling adults with a diagnosis of mild cognitive impairment or early-stage Alzheimer's disease, together with their live-in caregivers, were enrolled in the study. After a brief training, participants were asked to meditate for 11 minutes, twice daily for 8 weeks. Major outcomes included measures of perceived stress (Perceived Stress Scale), sleep (General Sleep Disturbance Scale), mood (Profile of Mood States), memory functioning (Memory Functioning Questionnaire), and blood pressure. Participants were assessed pre- and post-intervention. *Results*. Ten participants (5 of 6 dyads) completed the study. Treatment effects did not vary by participant status; analyses were thus pooled across participants. Adherence was good (meditation sessions completed/week: *X* = 11.4 ± 1.1). Participants demonstrated improvement in all major outcomes, including perceived stress (*P* < 0.001), mood (overall, *P* = 0.07; depression, *P* = 0.01), sleep (*P* < 0.04), retrospective memory function (*P* = 0.04), and blood pressure (systolic, *P* = 0.004; diastolic, *P* = 0.065). *Conclusions*. Findings of this exploratory trial suggest that an 8-week meditation program may offer an acceptable and effective intervention for reducing perceived stress and improving certain domains of sleep, mood, and memory in adults with cognitive impairment and their caregivers.

## 1. Introduction

Alzheimer's disease (AD), the most common form of dementia, is a chronic, progressive brain disorder resulting in a loss of memory, reasoning, language skills, and the ability to care for one's self [1]. AD is the seventh leading cause of death in the US [2], affecting 5.3 million Americans at an estimated cost of $148 billion, figures that are expected to increase dramatically in the coming years [3, 4]. AD affects quality of life for both the patient and the caregiver in profound ways. Many individuals with cognitive impairment become unable to engage in once loved activities that gave them a sense of purpose or pleasure [5]. Behavioral and social skills may also deteriorate, resulting in feelings of social isolation, anxiety, and depression, which, in turn, further increase risk for poor mental and physical health outcomes [6, 7]. For example, neuropsychiatric symptoms are common in adults with AD, as well as in those with mild cognitive impairment (MCI) [8, 9]. Up to 64% of AD patients [10] and 59% of those with mild cognitive impairment [11] suffer from sleep disruptions, and depressive symptoms are common in both AD and MCI sufferers, affecting up to 87% and 83% of these populations, respectively [12, 13].

Caregivers are also at elevated risk for distressful symptoms, including increases in sleep disturbances, depressive symptoms, and burden, as well as cognitive decline relative to age and gender-controlled noncaregivers [14, 15]. For example, studies in community-dwelling caregivers of persons with dementia have prevalence rates of self-reported sleep disturbances and depression of up to 68% and 55%, respectively [16, 17], as well as a six-fold increased risk of dementia compared to noncaregivers [18]. Likewise, recent research has shown caregivers of MCI patients to suffer significantly elevated burden [19] and mood disturbance [20], and to have a need for increased support services that is comparable to those caring for dementia patients [21]. Distressful feelings, impaired sleep, and subsequent maladaptive behaviors in caregivers have been associated with further deterioration of the care recipient's functional and psychological status, culminating in further distress in the caregiver [17, 22, 23]. Distressful sleep and mood responses of both members of the caregiving dyad can thus contribute to a vicious cycle that may lead to deleterious health effects for both, and, ultimately, to institutionalization of the care recipient [6, 17, 24, 25].

There is an obvious need to identify prevention and management strategies that target the complex effects of chronic stress and address the associated multiple, interrelated mental, and physical health challenges affecting these vulnerable caregiver dyads. Of particular promise in this regard is meditation, an ancient psychophysical discipline that is gaining increasing favor throughout the western industrialized world as a means of reducing stress and improving mental and physical well-being [26].

As indicated in recent systematic reviews by our group and other investigators, and by the growing body of original research on the health effects of meditation, there is mounting evidence that even brief (5 days −8 weeks) meditation programs may improve neuropsychological, metabolic, and clinical profiles in a range of populations [26–30]. For example, studies have shown meditation to reduce perceived stress [28, 31–33], anxiety [28, 31, 33], and depressive symptoms [33–35], enhance quality of life [30, 34], decrease sleep disturbance [32], improve several domains of cognition [35], reduce sympathetic activation, and enhance cardiovagal tone both acutely and long term in clinical as well as nonclinical populations [27, 36].

While research in Alzheimer's patients and their caregivers remains limited, findings from previous observational studies and a recent small clinical trial suggest that meditation practice may reduce stress, anxiety, and depression and improve health and cognitive outcomes in both adults with cognitive impairment and their caregivers [31, 37–39]. However, no studies to date have examined the effects of a structured meditation program in caregiver dyads. To our knowledge, the current pilot study is the first trial to investigate the effects of meditation on perceived stress and related indices of psychological morbidity and sympathetic activation in caregiver dyads, and among the first to investigate the effects of meditation in caregivers or AD patients.

## 2. Methods

### 2.1. Study Participants

Community-dwelling caregiving dyads were recruited using newspaper ads, flyers, and brochures placed in medical offices (e.g., the UVA Memory Disorders Clinic) and other public places in Charlottesville, VA, USA. Study advertisements detailed the study and eligibility requirements and provided study contact information for those interested in participating. Eligible participants were caregiving dyads composed of: (1) an adult with a physician-confirmed diagnosis of MCI or early stage AD of at least 6-week duration, and current examination within the last 12 months with a score of 20 or higher on the Mini Mental State Exam (MMSE); and (2) a live-in caregiving relative (both members of the caregiving dyad were required to enroll in the study); 18–100 years of age, English-speaking, and willing and able to complete paper-and-pencil questionnaires and abide by the protocol. Reasons for exclusion included: mid or late stage dementia; history of schizophrenia or psychosis; pregnant or caring for an infant; primary caregiver for a second person not in this study; began or stopped taking a cholinesterase inhibitor (e.g., donepezil (Aricept)) or psychotropic medication (e.g., antipsychotic and antianxiety agents) within the previous 6 weeks; serious physical trauma or diagnosis of serious chronic health condition requiring medical treatment and monitoring within the previous 3 months (e.g., diabetes, serious renal disease, and cancer); acute coronary syndrome or cerebrovascular event within the past 6 months (e.g., myocardial infarction, and coronary artery bypass); and meditation practice within the past 12 months. All participants provided informed consent, and the study was approved by the University of Virginia Institutional Review Board.

### 2.2. Outcome Measures

Assessment of perceived stress and related physiologic and psychological profiles was performed twice during the study: at baseline and following the 8-week treatment period. The baseline assessment was performed immediately following provision by the participant of written informed consent to participate in the study. The follow-up assessment was performed upon each participant dyad's completion of the 8-week meditation program. At each visit, heart rate and blood pressure, measures of sympathetic activation, were measured three times, and the average was recorded using an automated blood pressure monitor (Omron Model HEM-780) following a 5-minute seated rest period. Each participant also completed a short battery of established, well-validated self-report instruments to assess perceived stress (Perceived Stress Scale [PSS]) [40], mood and affect (Profile of Mood States [POMS]) [41], Positive and Negative Affect Scale [PANAS]) [42], stress hardiness (Dispositional Resilience Scale [DRS]) [43], sleep quality (General Sleep Disturbance Scale [GSDS]) [44], self-compassion (Self-Compassion Scale [SCS]) [45], and cognitive status (Memory Functioning Questionnaire [MFQ]) [46]. In addition, participants were administered a brief treatment expectancy questionnaire, as well as a short exit questionnaire adapted from that used in our previous studies regarding the effects of yoga on gait in the elderly [47] and cardiovascular disease (CVD) risk in older adults. This survey includes both structured and open-ended questions regarding the participants' experience with the study staff, perceived benefits and problems with the meditation intervention, reasons for leaving the study early or not adhering to the study protocol, and other concerns. Specific questions regarding perceived measurement burden were included. Participants completed the exit questionnaire at the follow-up assessment or (for those not completing the study), at another time of the participant's choosing. In addition, participants recorded the number of minutes of meditation practiced each day for the 8 weeks of the study using a daily meditation log.

### 2.3. Intervention

Immediately following baseline assessment, participants received 30–45 minutes of in-person meditation training. Participants were taught the Kirtan Kriya, a basic, easy-to-learn form of yogic meditation that incorporates both a mental component (repetition of the Sa-Ta-Na-Ma mantra) and a physical/motor component (touching the thumb to each fingertip in sequence with the mantra). For this study, we used the 11-minute version of the practice (repeating the mantra for: 2 minutes aloud, followed by 2 minutes whispering, 3 minutes silently, 2 minutes whispering again, and ending with 2 minutes aloud). Participants were given a meditation CD and an illustrated instruction sheet for home use. The meditation CD contained four tracks: the first track comprised an 11-minute guided meditation session which participants were instructed to follow at least once a week to reinforce the in-person training; the second track was identical to track one but accompanied by ocean sounds; the third track provided only the timing cues needed for the participants to conduct the meditation session without guidance; track four also provided only timing cues, but coupled with ocean sounds. Participants were instructed to meditate for 11 minutes twice a day, every day for 8 weeks (for a total of 112 sessions) and to record each practice session on the daily meditation log.

### 2.4. Statistical Analysis

Descriptive statistics were generated for the baseline characteristics of each group: cognitively impaired patients and caregivers. Potential differences between characteristics of caregivers and cognitively impaired participants were evaluated using chi square (for categorical variables), independent student's *t*-tests (for continuous variables with a normal distribution), or Wilcoxon signed rank (pre-post) tests (for ordinal variables or continuous variables with evidence of skewing). We used separate repeated measures ANOVA models (multivariate tests) to assess the effects of meditation on change over time (baseline to 8 weeks) in perceived stress and in related indices of psychological and physiological health. Because this was a small exploratory pilot study, and we were looking for trends as well as significant differences, all statistical tests were evaluated using an alpha of 0.05 (two-tailed test).

## 3. Results

Twelve adults (6 dyads), ranging in age from 48 to 85 years (*X* = 73.3 ± 3.9 years), enrolled in the study (Table 1). Seven participants were female (3 caregivers and 4 cognitively impaired), and 5 were male (3 caregivers and 2 cognitively impaired). All participants were married; five of the six caregivers were spouses. One participant with cognitive impairment was cared for by her daughter. Ten participants were retired, one (caregiver) was a homemaker, and one (caregiver) was employed full time. Ninety-two percent of participants were college educated, with 58% reporting a Bachelor's degree or higher level of education. All participants were non-Hispanic white. Poor sleep quality and/or daytime sleepiness/fatigue at least 2 times per week was reported by more than 90% of participants. Caregivers reported significantly poorer sleep (*P* < 0.01) and demonstrated significantly higher memory function (*P* = 0.01) at baseline than did participants with cognitive impairment but were similar in other baseline characteristics (Table 1).

Ten participants (5 dyads) completed the study, including 6 women and 4 men; one dyad withdrew in the first 2 weeks due to scheduling conflicts. Compliance was very good overall, with participants completing an average of 11.4 ± 1.1 meditation sessions per week (out of 14 possible). Because treatment effects did not vary by participant status (cognitively impaired versus caregiver), the two groups were pooled for the purposes of analysis. As illustrated in Table 2, participants demonstrated statistically significant improvement in the primary outcome measure, perceived stress (*P* = 0.03) as well as in sleep quality (*P* = 0.02), retrospective memory function (*P* = 0.04), and systolic blood pressure (*P* = 0.004) following the 8-week intervention. Participants also demonstrated significant or marginally significant reductions in diastolic blood pressure (*P* = 0.065) and mood impairment as measured by the POMS (overall, *P* = 0.07; depression, *P* = 0.01; anger/hostility, *P* = 0.09). Adjustment for treatment expectancy did not alter these findings. Reduction in perceived stress was correlated with positive changes in total mood (*r* = 0.83  and  *P* = 0.003) and sleep scores (*r* = 0.57  and  *P* = 0.08). Similarly, improvement in sleep was strongly correlated with improvements in mood (*r* = 0.71  and  *P* = 0.03), again suggesting strong inter-relationships among these factors. No statistically significant improvements in positive or negative affect (as measured by the PANAS) were noted (Table 2). In addition, participants did not show significant changes in either stress hardiness or self-compassion, suggesting that the observed improvements in stress, sleep, and mood were not mediated by these factors.

Participant feedback regarding the study and the meditation program was positive overall. Responses to structured and open-ended questions on exit questionnaires and comments on meditation logs indicated generally high satisfaction with the program, with 9 of the 10 participants who completed the exit questionnaire indicating that they enjoyed the meditation and the quiet time to relax and/or reflect. Concerns regarding the program included scheduling the time to meditate, with nine participants commenting that they had some difficulty with the time factor. One caregiver reported difficulty concentrating (but appreciated his wife's dedication and the obvious help it has been to her), and another caregiver stated that the cognitively impaired member of the dyad found the practice challenging.

## 4. Discussion

Findings of this preliminary pilot study suggest that a meditation program is feasible to implement in adults with cognitive impairment and their caregivers, and may offer a cost-effective intervention for improving perceived stress, mood, sleep, and blood pressure in this population. To our knowledge this is the first study to assess the effects of a mind-body program in caregiver-cognitively impaired dyads, and among the first to evaluate the effects of a simple meditation program in community-dwelling adults with cognitive impairment. Our findings are consistent with those of a recent study in 14 adults with memory loss that showed improved well-being and neuropsychological function over time, along with increased cerebral blood flow after a similar, 8-week, 12-minute/day Kirtan Kriya meditation program [39, 48]. In contrast to our findings, a recent controlled study of mindfulness meditation in caregivers of dementia patients did not show significant improvements over time in caregiver-perceived stress, mood, or sleep [49], possibly in part due to reduced compliance [4]. Studies regarding the effects of more complex mindfulness-based meditation programs in caregivers have also shown significant attenuation of most benefits over time [50, 51], perhaps in part due to the higher time demands of the intervention. A simpler meditation program such as that implemented in this study may carry advantages in terms of sustainability, especially in already heavily burdened populations such as caregivers. While it is unknown if the benefits observed in our study persisted, the high compliance and generally high satisfaction expressed by participants are encouraging. Based on participant comments, compliance and continued practice might be further improved by emphasizing flexible practice times to accommodate different or changing schedules.

Identifying feasible, cost-effective interventions for reducing stress and for improving sleep and mood in both cognitively impaired adults and their caregivers is of clear importance, given the high prevalence and negative impact of chronic stress, sleep disturbance, and mood impairment in these populations. For example, the chronic stress that often characterizes the lives of family caregivers has been linked to adverse changes in sleep [17], mood [52, 53], and immunological function [52, 54] and elevated risk for metabolic syndrome, CVD, and mortality [55, 56] in this population. Chronic psychological stress can have profound effects on memory and behaviors in persons both with and without cognitive impairment and has been prospectively linked to increased risk for dementia in older adults [57]. Elevations in hypothalamic pituitary adrenal (HPA) axis activity, manifested by elevated cortisol levels, are associated with hippocampal volume loss and memory impairment in nondemented, elderly persons [58, 59]. Further, in mouse models for Alzheimer's disease, studies show elevated production of *β*-amyloid under stressful conditions, suggesting that stress may contribute to an increase in plaque deposits and progression in AD [60].

Similarly, prevalence of mood disorders is high in both AD patients and their caregivers. For example, depressive symptoms are estimated to affect up to 87% and 55%, respectively, of these populations [12, 16]. Depressive symptoms and other distressful states have, in turn, been linked to significantly increased risk for diabetes, CVD, stroke [61], and other components of the metabolic syndrome [62, 63] and are a significant contributor to the profound reductions in quality of life reported by those with cognitive impairment and their family caregivers [12, 64]. In addition, mood disturbance can contribute to impairment of both sleep and memory, as well as to HPA axis dysregulation and autonomic dysfunction and related proinflammatory changes; in this way, poor emotional health may promote a vicious cycle of adverse physiologic, neuroendocrine, and psychosocial changes that foster the development and progression of CVD, AD, and related chronic conditions [59, 65, 66].

Sleep disruption, also common in cognitively impaired adults and their caregivers, likewise has negative effects on health, functioning, and quality of life in both patients and their carers and is a major reason for institutionalization [11, 24, 25]. Sleep disturbances have been strongly associated, in a bidirectional manner, with depression and other distressful states [67], autonomic dysfunction [68, 69], and can promote glucose intolerance, proinflammatory changes, dyslipidemia, obesity, and hypertension [68, 70, 71]. Sleep disturbances have likewise been linked with increased risk for both for incident type 2 diabetes and for CVD morbidity and mortality [59, 70–72]. The association of sleep to chronic illness and related risk factors appears strongly reciprocal [70, 73].

The reductions in blood pressure observed in this study are consistent with previous research regarding the effects of simple meditation programs in older adults with hypertension, coronary artery disease, and related chronic conditions [36, 74, 75]. Caregivers are at greater risk for hypertension [76], and recent research has suggested that elevated blood pressure may largely explain the increased coronary heart disease risk observed in this population [77]. Elevated blood pressure has also been linked to subsequent cognitive decline and implicated in the initiation and progression of AD [78–80].

Thus, if our findings are confirmed in larger randomized controlled trials, a simple and inexpensive intervention, meditation, may offer psychological, cognitive, and physiological benefits to both cognitively impaired adults and their caregivers, which in turn could have important implications for physical and mental health, emotional well-being, and cognitive function in both populations.

Strengths of the study include the community-based design, the inclusion of both cognitively impaired patients and their caregivers, and the high retention and compliance of participants. However, limitations of this pilot study are several. The sample size was small, limiting power and generalizability. The study lacked a control group, raising the possibility that our findings could be in part explained by a placebo effect. However, adjustment for treatment expectancy did not attenuate the observed improvements, suggesting that expectation of benefit did not account for the observed improvements. Participants were relatively well educated, most were retired, and all were non-Hispanic white, again limiting generalizability to other ethnic and socioeconomic groups. Our study sample comprised community-living dyads who were willing and able to participate in a meditation trial and thus are likely not representative of all cognitively impaired adults and their caregivers. The study was relatively short term and did not include a follow-up component, so persistence of benefits is unknown.

### 4.1. Conclusions

Findings of this exploratory trial suggest that a simple meditation program may offer an acceptable and effective intervention for reducing perceived stress and blood pressure, and improving certain domains of sleep, mood, and memory in adults with mild cognitive impairment or early stage Alzheimer's disease and their caregivers. These preliminary findings warrant confirmation in larger, controlled trials and in ethnically and socioeconomically diverse populations.

## Figures and Tables

**Table 1:** Baseline characteristics of participants with MCI/early stage Alzheimer's disease and their caregivers (*N* = 6 dyads).

	Cognitively impaired (*N* = 6)		Caregivers (*N* = 6)		*P*
	Mean/*N*	SE/percent	Mean/*N*	SE/percent	
*Demographic factors*					
Age (mean ± SE in years)	75.00	3.65	71.50	5.25	NS
Gender					NS
Male	2	33.33%	3	50.00%	
Female	4	66.67%	3	50.00%	
Education					NS
At least 4 years of college	4	66.67%	3	50.00%	
Less than 4 years of college	2	33.33%	3	50.00%	
Married	6	100.00%	6	100.00%	
Occupation					NS
Retired	5	83.33%	5	83.33%	
Homemaker	1	16.67%	0	0.00%	
Employed	0	0.00%	1	16.67%	
*Mood, stress, and sleep quality*					
Perceived stress scale	17.33	2.95	17.33	2.20	NS
Profile of mood states (POMS)					
Total	17.50	8.37	18.17	7.60	NS
Tension/anxiety	4.33	2.11	6.33	1.61	NS
Confusion	6.67	1.28	3.17	1.78	NS
Depression	9.50	3.08	7.83	2.56	NS
Anger/hostility	7.67	2.19	7.00	1.24	NS
Vigor	17.33	2.16	16.17	1.58	NS
Fatigue	6.67	1.78	10.00	0.97	NS
Positive-negative affect scale					
Negative affect	15.33	1.67	15.33	1.56	NS
Positive affect	33.50	2.53	34.00	2.57	NS
General sleep disturbance scale (Total)	19.33	3.73	49.83	7.05	0.003
*Memory functioning scale (Total)*	230.67	6.78	304.50	13.73	0.001
*Measures related to sympathetic activation*					
Heart rate (average)	65.67	3.68	63.83	5.48	NS
Systolic blood pressure (average)	129.83	9.02	132.83	6.33	NS
Diastolic blood pressure (average)	72.17	5.80	78.00	3.06	NS

NS: *P* > 0.10.

**Table 2:** Change over time in indices of psychosocial status, sleep, memory functioning, and sympathetic activation in adults with cognitive impairment and their caregivers (*N* = 10 participants).

	Baseline	Posttreatment	P*
	(Mean ± SE)	(Mean ± SE)	
*Mood, stress and sleep quality*			
* Perceived stress scale*	17.3 ± 2.1	11.8 ± 1.4	0.03
* General sleep disturbance scale*			
* Total*	32.9 ± 5.8	26.9 ± 3.8	0.02
Daytime fatigue	7.1 ± 1.7	4.9 ± 0.7	0.04
Daytime alertness	15.3 ± 1.8	14.4 ± 1.6	NS
Sleep duration	8.6 ± 2.1	5.8 ± 1.9	0.04
Sleep medication	5.7 ± 1.9	5.2 ± 1.8	NS
* Profile of mood states*			
* Total*	17.9 ± 6.5	9.7 ± 4.1	0.07
Tension/anxiety	5.4 ± 1.5	4.7 ± 1.7	NS
Confusion	5.3 ± 1.4	4.5 ± 1.2	NS
Depression	9.4 ± 2.2	5.7 ± 1.2	0.01
Anger/hostility	7.2 ± 1.4	6.0 ± 1.4	0.09
Vigor	16.8 ± 1.4	18.1 ± 1.7	NS
Fatigue	7.4 ± 1.1	6.9 ± 0.9	NS
*Memory functioning scale*			
* Total*	255.1 ± 13.9	252.0 ± 12.2	NS
Frequency forgetfulness	140.1 ± 10.1	134.4 ± 8.2	NS
Seriousness of forgetting	78.9 ± 5.9	75.4 ± 5.8	NS
Retrospective memory functioning	10.8 ± 1.8	17.1 ± 2.5	0.04
Mnemonic	25.4 ± 3.0	25.1 ± 1.9	NS
*Measures related to sympathetic activation*			
Heart rate (average)	64.0 ± 1.5	63.8 ± 1.6	NS
Systolic blood pressure (average)	128.2 ± 4.0	121.8 ± 4.0	0.004
Diastolic blood pressure (average)	74.1 ± 1.9	69.4 ± 2.0	0.07

*Repeated measures ANOVA.

NS: *P* ≥ 0.10.
